# Evidence, ethics and the promise of artificial intelligence in psychiatry

**DOI:** 10.1136/jme-2022-108447

**Published:** 2022-12-29

**Authors:** Melissa McCradden, Katrina Hui, Daniel Z Buchman

**Affiliations:** 1 Joint Centre for Bioethics, University of Toronto Dalla Lana School of Public Health, Toronto, Ontario, Canada; 2 Bioethics, The Hospital for Sick Children, Toronto, Ontario, Canada; 3 Genetics & Genome Biology, Peter Gilgan Centre for Research and Learning, Toronto, Ontario, Canada; 4 Everyday Ethics Lab, Centre for Addiction and Mental Health, Toronto, Ontario, Canada; 5 Department of Psychiatry, University of Toronto, Toronto, Ontario, Canada

**Keywords:** Psychiatry, Decision Making, Ethics- Medical, Mental Health

## Abstract

Researchers are studying how artificial intelligence (AI) can be used to better detect, prognosticate and subgroup diseases. The idea that AI might advance medicine’s understanding of biological categories of psychiatric disorders, as well as provide better treatments, is appealing given the historical challenges with prediction, diagnosis and treatment in psychiatry. Given the power of AI to analyse vast amounts of information, some clinicians may feel obligated to align their clinical judgements with the outputs of the AI system. However, a potential epistemic privileging of AI in clinical judgements may lead to unintended consequences that could negatively affect patient treatment, well-being and rights. The implications are also relevant to precision medicine, digital twin technologies and predictive analytics generally. We propose that a commitment to epistemic humility can help promote judicious clinical decision-making at the interface of big data and AI in psychiatry.

## Introduction

There is considerable excitement about the promises of improving healthcare delivery and health systems with artificial intelligence (AI) and machine learning (ML). AI (In this paper, we use the term AI to encompass a broad range of algorithmic systems including ML, deep learning and where these systems may be both supervised and unsupervised) in healthcare generally refers to a system which computes live, incoming data to generate predictions in real time for patients. ML refers to a branch of methodologies used to achieve this functionality through the development of algorithms. Proponents suggest that leveraging big data (including genomics, demographic and environmental information) can improve access, diagnostic accuracy, guide prognostication, discover new treatments and provide more efficient and higher quality patient care. While research into the potential psychiatric applications of AI are in the nascent stage,^1^ researchers are studying how electronic health records (EHR), rating scales, brain imaging data, social media platforms and sensor-based monitoring systems can be used to better predict, classify or prognosticate mental illnesses such as depression and psychosis,^2 3^ or predict the risk of suicide.^4^

Much has been written about the ‘biomedical aspirations of psychiatry’^5^ and the decades-long ‘crises’ of uncertainty regarding diagnosis, aetiology and treatment.^6–8^ Accordingly, it is foreseeable that some clinicians may view the advances of AI in psychiatry as a corrective to the ‘uncertainty work’^9^ that characterises everyday practice and perhaps medicine more generally.^10^ Given the desire to promote the well-being of their patients, some clinicians may perceive an epistemic obligation to align their clinical judgements with the algorithmic outputs in the interest of high-quality evidence-based decision-making.^11 12^ The hope is that AI and digital technologies will help promote improved access to treatment and quality of care.^13^ Early work has focused on tools like conversational AI (ie, chatbots) to provide cognitive behavioural therapy and more integrated digital care delivery systems, both of which remain in their infancy and have been met with challenges with uptake and implementation.^1 14 15^

While AI systems create challenges and opportunities for clinical decision-making in psychiatry, they also reveal the entanglement of epistemology and ethics. For example, evidence-based improvements to clinical outcomes using AI remain limited^16^ and AI’s ability to provide individual-level insights via explanation (eg, identifying individual patient features driving specific outcomes) is highly contested.^17 18^ Additionally, some scholars have highlighted how premature optimism surrounding the perceived epistemic superiority of AI to guide clinical judgements may entrench systems of power within healthcare.^19^ This may further intensify structural vulnerabilities of some patient populations—such as people living with mental illnesses—which may further shift epistemic power away from these groups.^20^ These concerns suggest that an epistemically humble approach to clinical decision-making is needed that balances relevant clinical and non-clinical information (including patient experiential knowledge) with a critical reflection on the limits of clinicians’—and the AI systems’—content expertise.

In this paper, we consider the potential implications of epistemically prioritising AI in clinical decision-making in psychiatry. We focus on psychiatry as a case example as potential consequences in this context are not trivial; some AI predictions may contribute to unnecessary institutionalisation, undermine patients’ credibility about their own experiences, and in extreme cases, contribute to decisions to remove a patient’s right to make their own treatment decisions. We proceed with our argumentation as follows. First, we explore the intersection of evidence-based medicine (EBM) with clinical judgement and AI. Second, we critically interrogate whether psychiatry can be ‘explained’ with ML. Third, we explore potential unintended consequences of AI in psychiatry and focus on AI as (perceived) expert and epistemic injustice and shared-decision making (SDM). Finally, we argue that to achieve maximum benefit of AI applications, clinicians’ out to commit to epistemic humility to support clinical judgements and SDM.

## EBM, clinical judgement and AI

There is a long-standing assumption that a researcher, as well as clinician, should approach science, or their patient, free from any subjective influences that could introduce bias and compromise the objectivity of the data and decisions.^21^ This is the logic of EBM. In response to decades of shifting opinions about psychiatric categorisation, the Diagnostic and Statistical Manual of Mental Disorders was created to standardise psychiatric practice and to systematise the classification of mental disorders. Nonetheless, some scientists and clinicians believe that psychiatric diagnoses represent heterogeneous presentations, such that two people, for example, could receive the same diagnosis without having any overlap in symptoms.^22^ Others suggest that clinical judgement in psychiatry undermines objectivity in an EBM paradigm. For instance, clinicians rely heavily on subjective factors such as patient testimony to make determinations such as psychiatric diagnoses, and sometimes the patient’s account does not align with clinicians’ assessments of symptoms or behaviours. Practice in psychiatry may not always resonate with EBM assumptions (eg, about the nature of disease or the diagnostic categories), such that clinical decision-making is often characterised by uncertainty involving imperfect information and incomplete data.^23^ While revised EBM models suggest integrating evidence with patient values and context,^24^ the objective uncertainty is a primary reason why psychiatry historically has been considered less scientifically rigorous by its biomedical counterparts.

Over the last century, there have been movements within psychiatry to promote data-driven, statistical and algorithmic approaches to clinical judgement that attempt to eliminate or carefully control confounders, values and bias.^25^ For example, there has been considerable effort and resources put toward identifying neuroimaging-based biomarkers and reconceptualising psychiatric disorders via new transdiagnostic frameworks like the Research Domain Criteria^26^ to improve explanations of psychopathology and to better target and tailor treatments. Despite the best efforts, identifying reliable biomarkers of psychiatric disorders remains a challenge.^27^

The notion that AI might catalyse a more reliable taxonomy of psychiatric disorders, as well as provide better predictions for people with–or who may develop–mental disorders is attractive.^1^ Providing a technological explanation of something as aetiologically and socially complex as mental disorder provides a sense of objectivity and value neutrality.^28–30^ Indeed, clinicians ought to apply the highest quality scientific evidence to support clinical decision- making. Given the power of AI to draw from and analyse tremendous amounts of information per second, clinicians may feel obligated to align their clinical judgements with the algorithmic outputs because the supposedly reliable scientific processes informing the algorithm should warrant high levels of confidence in decision-making.^12 31–33^ Furthermore, some clinicians may have liability concerns if they do not follow the recommendation of an algorithmic system that contradicts their clinical judgement, a pressure that may increase should the use of AI tools become the standard practice in the future.^31 34 35^ The supposedly impartial, objective, and therefore, superior AI process should enable clinicians to enact their fiduciary duty of promoting the best interests of their patient.

Clinicians are keenly aware of the challenges to diagnostic and prognostic accuracy and any tool to improve that knowledge can provide some solace to their clinical judgements and SDM processes. At this time, there is limited research on how AI might influence SDM.^36 37^ SDM is ‘an approach where clinicians and patients share the best available evidence when faced with the task of making decisions, and where patients are supported to consider options, to achieve informed preferences’.^38^ SDM is considered a key component of high-quality patient-centred care.^39^ However, some scholars argue that AI could have a ‘third wheel’ effect on the SDM process. Triberti *et al* 36 postulate this effect could manifest in three ways: (1) clinical decisions could be delayed or come to a standstill when AI-generated recommendations are difficult to understand or explain; (2) patients’ symptoms and diagnoses could be misinterpreted when clinicians attempt to fit them into existing AI classifications, resulting in an erosion of trust or potential epistemic injustice (see Epistemic Injustice, AI and SDM below) and (3) confusion as to whether the algorithmic output or clinician has epistemic authority over treatment recommendations, and how any ambiguity might be negotiated.^36^

Birhane^40^ notes that relying on ML-generated predictions is particularly tenuous in contexts such as psychiatry where considerable complexity and ambiguity characterise the taxonomies. Indeed, scholars are split with respect to the potential automated future of psychiatry. Some argue that AI is no different from the multitude of tools clinicians employ to measure a patient’s experience and support SDM; they are helpful towards their purpose of measurement, but always require context for interpretation. Others suggest that the (expected) superiority of AI tools in psychiatry to diagnose and make treatment recommendations will become strongly preferred to humans, so patients can be ‘treated to the best of scientific understanding’.^41^

## ‘Explaining’ psychiatry with ML?

Given that the precise mechanisms giving rise to psychiatric disorders are highly complex, some have proposed that AI offers greater certainty and the potential to illuminate previously unknown relationships between symptoms and treatments, disease clusters and genetics.^42^ Explainability—a suite of methodologies enabling transparency by revealing a model’s operations—has been posited to reveal these insights. We argue that explainability parallels modern historical trends in psychiatry which strive to identify more objective approaches to diagnosis, prognosis and treatment.

Explainability can be divided into inherent (ie, interpretability, revealing the model’s workings as a system) and post hoc explainability (using a secondary algorithm to reveal the ‘reasons’ behind an individual-level prediction).^18^ Some argue that explainability—and post hoc explainability in particular—has immense ethical value, and is instrumental to informed consent, responsible clinical decision-making and medicolegal accountability.^43 44^

While these goals are laudable, explainability’s reliability and ethical significance has been called into question.^45–47^ Inasmuch as clinicians believe that post hoc explanations can provide the reasons behind the prediction for an individual patient, current explainability methods simply cannot deliver in this regard.^18 48^ Ghassemi *et al* suggest that, at present, there is no computationally reliable way to discern whether the explanation one receives is specific to the patient in question or referring to the more general operations of the algorithm.^18^ The implication is that when a clinician looks to an explanation behind a patient’s prediction, they cannot be assured that the model is computing that individual patient’s features, versus whether it is deriving explanations based on the model as a whole. This means that there is no reliable way to know whether the model’s explanation is specific to the patient or is in fact a general explanation.

In psychiatry, there may be unique challenges relating to the verification of a prediction’s accuracy and individuality. For example, when the output of a saliency map intended to explain a diagnosis of pneumothorax highlights an area of the shoulder, it is readily spotted as an error.^49^ But if the explanation for a patient’s predicted suicide risk is the feature ‘history of suicidality,’ there is no objectively verifiable means of assuring oneself that it is this history which is a clinically significant contributor to this patient’s present state. Similarly, a prediction of which therapist a patient will most benefit from^50^ could be accompanied by a list of features for why the model has made this prediction; yet, we cannot be assured that it is these features which independently influence the beneficial treatment response observed, nor are they a guarantee of such.

These technical limitations are presently underappreciated. This is problematic given that recent work notes that clinicians tend to view explanations ‘as a means of justifying their clinical decision-making’.^51^ Emerging evidence indicates an exacerbation of automation bias—a well-characterised problem over over-reliance on computational systems^52^—with AI systems. Particularly, the output of an AI system, even when wrong, may mislead some clinicians to follow its recommendation even against their initial (and perhaps accurate) judgement.^53–56^

## Potential unintended consequences of AI in psychiatry

Despite the best intentions of AI developers, decision-makers and clinicians, there may be unintended consequences associated with implementing AI in psychiatry.

### AI as expert

The field of computer science has long considered AI to be an expert system, which is a programme that simulates the judgements and behaviours of a human expert.^57^ If AI predictions are considered to produce knowledge superior to that of expert clinicians, this means that the predictions rank higher on an epistemic hierarchy than other forms of knowledge, such as professional clinical judgement and patient experiential knowledge.^58^ In other words, the algorithmic outputs have expert status.

The relationship between technology and expert status is not a new idea in medical sociology, as there are implicit rankings of various medical technologies (ie, drugs, devices and procedures) which provide more credibility on those who use the higher ranked technologies.^59^ For example, in one survey on public attitudes toward robotic surgery, over half of respondents indicated they thought hospitals that had a surgical robot were ‘better’ than those without.^60^ Indeed, clinicians have expert status, and, through their years of education, experience and training, have been given the social warrant to decide how a psychiatric condition should be understood and managed.^59^ Furthermore, the presumed expertise of AI systems and the widespread promotion of AI as ‘technical solutionism’—that new technologies can solve complex socio-technical problems^61^—may enhance the perceived credibility of AI systems in clinical decision-making and the power and credibility of clinicians and institutions who adopt AI systems into their workflow.^20^

As an entity contributing to the knowledge that forms the basis for making ‘good’ clinical decisions, even a perfectly reliable AI system is not determinative. While an AI can be developed and validated to the point where it is highly reliable at accomplishing a specific task, often this task itself is but a subset of the considerations necessary to make a good decision.^62^ For example, Jacobs *et al* note that clinicians felt an algorithm designed to predict drop-out risk for antidepressant medications could be highly useful while still representing only a subset of the considerations needed to prescribe a medication.^63^

### Epistemic injustice, AI and SDM

Despite the good will of clinicians, the potential expanded apparatus of AI systems in psychiatry may unintentionally create a harm called epistemic injustice. Epistemic injustice is a type of harm done to individuals or groups regarding their ability to contribute to and benefit from knowledge.^64^ Epistemic injustice occurs in two situations. First, testimonial injustice arises when a speaker (eg, a patient) is not believed or is taken less seriously than they deserve.^64^ For example, stereotypes of people with mental illness such as being cognitively unreliable or emotionally unstable often encourage others, including clinicians, to consider their testimonies as irrelevant, confused or time-consuming.^65 66^ Second, hermeneutical injustice arises when a person is at an unfair disadvantage because they, including the society they are in, lack the concepts to understand and communicate their experiences. Hermeneutical injustice is influenced by societal norms and the privileging of certain types of information.^67^ Fricker provides the example of sexual harassment—a concept that did not exist until the 1970s. If a person experienced sexual harassment in the workplace pre-1970s, they might interpret unwanted sexual advances as socially acceptable behaviour (eg, ‘mere flirting’). Hermeneutical injustice can arise in psychiatry if patient experiences are forced into an established diagnostic framework that may limit their ability to understand and frame their experiences in ways that might be meaningful to them.^66^

If the AI system is informed, for instance, by digital phenotyping data that uses natural language processing such as sentiment, lexical and semantic analysis from smartphones and social media,^3^ testimonial injustice could arise if the algorithmic information is considered a superior way of knowing and the patient’s subjective self-report is treated as lower-ranked evidence in clinical decision- making (see box 1).^32^ Indeed, a recent scoping review on the application of AI in SDM in healthcare identified a lack of focus on patient values and preferences.^37^ Furthermore, datasets used to train the AI may also themselves be biased based on the quality of diagnostic labels used to train the system,^3^ stigmatising descriptors in the EHR,^68^ as well as more severe presentations in under-represented groups due to upstream barriers to accessing care. Dataset bias introduces testimonial injustice by reifying labels that contribute to the downgrading of patient testimony.

Box 1 **Hypothetical clinical scenarios illustrating potential unintended consequences of artificial intelligence in psychiatry**

A patient comes into the psychiatric emergency department requesting treatment for their highly distressing suicidal thoughts, low mood and anxiety. A model designed to predict acute risk to prioritise patients for urgent care predicts a low likelihood that this patient is acutely in need based on a previously documented diagnosis of borderline personality disorder with minimisation of stated reasons that put their risk above their baseline and the resulting action is to refer them to their outpatient providers.

This situation indicates a form of testimonial injustice because a patient’s overt request for help is being denied because of an algorithmic prediction. Essentially, the model’s verdict is valued higher than the patient’s report, who is requesting urgent care. It is also true that there are cases where urgent care is not the appropriate venue in which to receive needed care; however, this decision should be made based on a clinical evaluation of the patient, not by the output of a model alone.

A patient is undergoing surgery for which postoperative opioid therapy is indicated. A machine learning (ML) system built into the jurisdiction’s prescription drug monitoring programme (PDMP) is designed to assess risk of opioid use disorder (OUD) and predicts she is at high risk. The physician, concerned about OUD, states they will not provide her with opioid treatment. The patient objects, noting that she has chronic pain secondary to endometriosis, which is greatly ameliorated by opioid medication when needed. She has managed her condition without issue for over a decade, and nothing else helps her pain. The physician knows the algorithm is widely used in practice and so the physician assumes that the patient is an unreliable narrator and declines to offer an opioid prescription.

Testimonial injustice in this case is reflected in the downgrading of the patient’s account, solely based on the ML prediction. In some jurisdictions in the USA, ML systems that generate risk scores are built into the PDMP, so some physicians may be legally required to consult the PDMP or risk losing their licence.^35^ This places an added pressure on prescribers to prioritise the ML verdict over what their patient is telling them, thereby potentially committing a testimonial injustice. As with all cases of testimonial injustice, those who are likely to be harmed disproportionately are patients who are members of structurally vulnerable populations.

A patient has agreed to an application wherein an AI system captures data from their social media activity to detect suicidality and can trigger an alert to their psychiatrist. After a tough day watching news stories, the patient posted a link to a news article with the comment ‘brb, jumping off the balcony now’. The app triggers the psychiatrist, who asks the patient to come in for an assessment. The patient reports they do not feel suicidal; the clinician feels they have an obligation to obtain a mental health assessment because of the app’s detection of suicidal ideation and an application for a mandatory psychiatric evaluation at a hospital initiated.

The clinician’s pursuit of a mandatory psychiatric evaluation in this case prioritises the model’s form of evidence regarding the patient’s mental health state over the patient’s own self-report. There is an understandably strong motivation to prevent suicide; however, to disregard a patient’s disclosure takes a presumptive view that patients are unreliable, and the cost of suicide is worth interfering with their liberty. These costs can be merely inconvenient (eg, having to receive a phone call when one is not actually suicidal) to significant (eg, having to present oneself for a mandatory psychiatric assessment). This scenario is a form of testimonial injustice that may undermine the trust between psychiatrists, the healthcare systems and patients.

Society is only beginning to make sense of the harms experienced via algorithms within and external to medicine. Many of us do not have the language to understand and communicate our experiences when algorithmic harm occurs. Noble describes a concept called technological redlining, which is the way digital data is used to create and sustain inequities between racialised and low-income populations.^69^ The term comes from the concept of redlining in housing discrimination, where in the 1930s banks withheld mortgages from customers who resided in or near predominantly Black neighbourhoods.^70^ Technological redlining may be a form of hermeneutical injustice. Hermeneutical injustice could arise through algorithmic classification of psychiatric diagnoses and predictions of likelihood to benefit from treatment which may influence care pathways and inadvertently widen inequities in access to and quality of care.

The potential uncritical prioritising of AI systems in psychiatric clinical decision-making creates a climate conducive to epistemic injustice. While explainable models may be perceived to satisfy ethical requirements for use, the knowledge of why a given prediction was generated is not akin to the knowledge that the use of that prediction will benefit an individual. For example, although there is imperfect knowledge in psychiatry regarding precisely how psychoactive medications give rise to their therapeutic effects, their use is justified by evidence collected through prospective clinical trials in a relevant patient population.^47^ To satisfy the ethical requirement for informed consent, clinicians should have knowledge of the conditions under which the model was evaluated in a clinical population. To point to model explainability as how clinicians satisfy informed consent poses a risk of exacerbating the power differential between clinicians and patients by prioritising knowledge of the model over the overall justification of the clinician’s judgement. Informed consent can be satisfied by the clinician conveying the evaluation of the AI system and explaining the rationale concerning how they are using the prediction to inform their recommendation. While many patients report that they are often not provided with the rationale behind existing treatment decisions or feel competent to challenge them,^67^ the use of AI in context may inadvertently exacerbate this harm. This includes situations where presumed objective algorithms might be used to justify more invasive technological surveillance over the daily lives of some populations, such as people with mental illness and who use drugs, who are already subject to high levels of surveillance by medical professionals and law enforcement.^20 71^

## AI-supported clinical judgement in psychiatry requires epistemic humility

We consider how to balance the anticipated benefit of psychiatric applications of AI with the need to promote epistemic humility in clinical judgements. Epistemic humility is a disposition as well as a commitment. It is an acknowledgement that AI in healthcare, inclusive of psychiatry, is accompanied by limitations of applying scientific knowledge to clinical decision-making, and that decisions are tentative and subjected to ongoing revision based on additional data as well as other contextual considerations. Being epistemically humble requires balancing scientific evidence, professional judgement and patient experiential knowledge.

While epistemic humility is a virtue individual clinicians should cultivate, it is also a characteristic of claims.^33^ The belief in the quality of the evidence is important, namely the scientific processes that produced the evidence leading to the algorithmic output. However, this ‘mechanical objectivity’,^72^ results in a kind of epistemic trust in mechanical procedures versus trust in scientists or its institutions. As described earlier, there are many concerns with the quality of evidence used to inform AI systems in healthcare, including psychiatry.

Epistemic humility reflects a situation where AI tools are applied, but the testimonies of patients ‘are recognised, sought ought, included within epistemic consideration, judged to be relevant and articulate (where they are) and, at least in certain respects, judged as epistemically authoritative’.^73^ We emphasise the ‘sought out and included within consideration’ because many patients, particularly those made vulnerable by systems of power (eg, racism, oppression, poverty, ableism) do not feel they are able to voice their perspectives during clinical encounters. For example, a patient (box 1) who is deprioritised for acute care may feel even more distressed and concerned that their thought processes appear so much more extreme than the assessment. They may feel powerless to dispute the assessment as they then question even their own reliability. To strive for epistemic humility, the clinician would accept the patient’s stated distress and either admit to hospital despite the prediction (should resources allow) or continue with an outpatient referral but with additional support. If the clinician must discharge the patient, they could arrange for follow-up phone calls to check in, adjust medication to address residual distress, and facilitate and expedited access to outpatient care. In contrast, rejection of epistemic humility would mean the acceptance of the algorithm’s prediction without further exploring the potential mismatch between its computations and the patient’s own assessment of their mental state.

Patient testimonies should not require validation by an AI system.^20^ Like Birhane,^40^ we do not dismiss the notion that AI predictions are meaningless because they neglect to understand lived experience. Rather, we caution that the anticipated uses of AI in psychiatry could have unintended consequences that are ethically important to address. Furthermore, more meaningful representation of medical knowledge related to the larger clinical picture can promote consistency in care, minimise medical errors, improve diagnosis and improve the quality of decision-making.^74^ For instance, psychiatric diagnoses can be biased by factors like race and ethnicity and AI may help eliminate these individual-level inconsistencies.^75^ Furthermore, AI could potentially be scalable such that large numbers of people could be screened in a cost-effective way.^76^ But to prevent the overgeneralisation of AI’s role in clinical decisions in psychiatry, it is important to be cautious against the potential epistemic privileging of AI in clinical judgements in psychiatry.^30^ We must move toward a model where AI can be incorporated into more nuanced and collaborative discussions with patients rather than a tool that could potentially supersede individual experiences, values, and preferences and reinforce existing power hierarchies at the expense of patient subjective experiential knowledge.^20 59^

### Humanistic practice of AI-inclusive psychiatry

We, like others, hope that any potential integration of AI into healthcare, including psychiatry, would help enrich its overall humanistic practice. We believe that most clinicians want to spend more time with their patients and take their patients’ testimonies seriously. Everyday pressures such as time, financial incentives and wait-list management make achieving this quite challenging. Indeed, freeing healthcare professionals from these burdens is what many hope AI integration in healthcare will help achieve.^77 78^ A great deal of evidence suggests that at least some of the benefits patients’ experience may be catalysed by a positive bedside manner, good communication and expectancy effects.^79–81^ Patients are more forthcoming, willing to pursue healthcare and overall participate in their care when they feel safe in healthcare settings. Many patients currently do not feel such safety for the reasons (and others) we have outlined above. To use AI to enhance medical knowledge without a concomitant enhancement of medical care would stunt its potential benefit to patients.

## Conclusion

In this paper, we argued that a potential epistemic privileging of AI in clinical judgements may lead to unintended consequences. The key to clinical decision-making grounded in epistemic humility requires clinicians to critically consider what goals are trying to be achieved by relying on the AI output before potentially relevant and legitimate perspectives offered by patients are deprioritised in clinical judgements. It is imperative that health systems that adopt AI-based predictions do not prioritise these outputs to the exclusion of SDM and incorporation of patient experiential knowledge.

In making our arguments, we are not privileging human clinical judgement over AI, claiming that AI is superior to clinical decision-making in psychiatry, or arguing categorically that AI does not have a role in augmenting clinical decision-making in psychiatry. Rather, we are concerned with AI’s potential place on the epistemic hierarchy in clinical decision-making. We argue that an uncritical acceptance of AI as being superior to humans in terms of accuracy, reliability and knowledge risks entrenching many of the inequities people living with mental illnesses have experienced for centuries. AI developers ought to be aware of the potential unintended consequences of their algorithms,^74^ and together with clinicians should work collaboratively with people with mental illness to develop–and access–the resources to understand and communicate their experiences of mental illness in the context of AI. This will help support health systems and clinicians commiting to epistemic humility in practice.

## Data Availability

No data are available.
